# Disease proportions and drug prescribing pattern observed in a free health camp organized at Dhorphirdi Village Development Committee of Western Nepal

**DOI:** 10.1186/s13104-015-1508-y

**Published:** 2015-09-29

**Authors:** Raj Kumar Thapa, Parbati Thapa, Kalpana Parajuli-Baral, Gulam Muhammad Khan

**Affiliations:** School of Health and Allied Sciences, Pokhara University, Lekhnath-12, Kaski, Nepal

**Keywords:** Disease, Drug prescribing pattern, Health camp, Nepal

## Abstract

**Background:**

Health camp is generally organized to provide health care services to the people deprived of health care facilities. The aim of this project was to assess the proportions of disease among attendees of health camp and study the drug prescribing pattern in a free health camp.

**Methods:**

A case study was performed from 1 day health camp to determine the proportions of disease and drug prescribing pattern. Data collection was performed using log book maintained in the health camp and patient’s demographic details, disease diagnosed and drug prescribed was obtained from same log book.

**Results:**

A total of 317 patients were included in the study. The majority of the patients were in the range of 41–50 years. On the basis of study on ethnicity, Brahmins and Chettris, were found to be predominant ethnic groups with gastrointestinal disorders as the major disease. The total number of medications prescribed was 510, with non-steroidal anti-inflammatory drugs (NSAIDs) and antipeptic ulcer drugs being commonly prescribed. The average number of drugs per prescription and the percentage of antibiotics prescribed were 1.6 and 21.4 %, respectively. It was observed that 96.8 % of prescription was by generic names. Likewise, 100 % of prescription included drugs from essential drug list.

**Conclusion:**

Majority of the patients were of working age group. Headache and fever were found to be the most prevalent cases and NSAIDs were the most commonly prescribed medications. The drug prescribing pattern of the free health camp complied with WHO recommended prescribing indicators.

## Background

The rational use of drugs requires that “patients receive medications appropriate to their clinical needs, in doses that meet their own individual requirements for an adequate period of time, at the lowest cost to them and their community” [1]. In 2010, the WHO reported that nearly half of the medicines are used inappropriately. Irrational use of drugs occurs universally and has become a global healthcare problem [2]. The WHO has suggested several indicators for evaluating the quality of drug use at health facilities. Some prescribing indicators include; average number and types of prescribed drugs, percentage of antimicrobial drugs, percentage of drugs prescribed by generic name and from essential drug list [3].

Health Camp is generally organized to provide health care services to the people who are generally out of reach to the basic health care facilities. Disease prevention as well as treatment of medical issues should be the main objective of any kind of health camps [4]. Prevalence of disease in particular area may be affected by various factors including socioeconomic status, life style, awareness among people, availability of health care facilities, etc. The types of drugs prescribed by doctors play a vital role in success of the prevention or treatment of a disease. Hence, the objective of this study was to determine the proportions of disease among attendees of health camp and drug prescribing pattern observed in 1 day free health camp organized at Dhorphridi Village Development Committee (VDC) of Western Nepal.

Free health camps are being organized by different organizations in different parts of the country. A study on drug prescribing pattern in a particular community might be helpful to organize a health camp where the participants could benefit the most. Additionally, such studies provide useful guidance to the organizers of health camp because organizing the health camp and evaluating its outcome is crucial and very few results regarding the use of medication in the health camp has been reported.

## Methods

### Study population

All the patients attending the health camp were included in the study. A total of 317 patients were assessed.

### Study design

The protocol of the study was approved by Research Committee of School of Health and Allied Sciences, Pokhara University and Helsinki ethical guidelines were followed during the entire study. A case study was conducted using the information of the free health camp program organized by School of Health and Allied Sciences, Pokhara University, Kaski, Nepal. The health camp was conducted at Dhorphirdi Village Development Committee of Tanahun district of Nepal with a team of 6 medical doctors, 6 registered pharmacists and 36 pharmacy students (Bachelor of Pharmaceutical Sciences), who participated voluntarily in the program.

The medications for the health camp were donated by Nepalese Pharmaceutical Industries, wholesalers and some retail pharmacies. Most of the drugs were dispensed from camp itself and only few medications which were not available were requested to purchase from outside. The medication were prescribed by the medical doctors and dispensed by pharmacists. The dispensing of medications was performed in a homemade paper envelope and all the necessary information like name of drug, dose, frequency, time, before or after food was delivered verbally as well as in written form. As a part of counseling, cross questioning was conducted to ensure they received correct information.

### Data collection

The data was collected from the log book maintained by the registered pharmacists in the health camp, which was initially prepared to enter all the necessary information like patient’s name, age, gender, ethnicity, disease diagnosed and drugs prescribed. Informed verbal consent was obtained from patients before enrolling them in the studies.

### Prescribing indicators

The WHO prescribing indicators were used to assess the appropriateness of prescribing pattern [1].

The prescribing indicators measured in this study were

1. The average number of drugs prescribed per encounter: calculated to measure the degree of polypharmacy. It was calculated by dividing the total number of different drug products prescribed by the number of encounters.
2. Percentage of drugs prescribed by generic name: calculated to measure the tendency of prescribing by generic name. It was calculated by dividing the number of drugs prescribed by generic name by total number of drugs prescribed, multiplied by 100.
3. Percentage of encounters in which an antibiotic was prescribed: calculated to measure the overall use of commonly overused forms of drug therapy. It was calculated by dividing the number of patient encounters in which an antibiotic was prescribed by the total number of encounters, multiplied by 100.
4. Percentage of drugs prescribed from an essential drug list (EDL): calculated to measure the degree to which practices confirm to a national drug policy as indicated in the national drug list of Nepal. Percentage was calculated by dividing number of products prescribed which were in essential drug list by the total number of drugs prescribed, multiplied by 100.

### Data analysis

Data were analyzed using SPSS (Statistical Package for Social Sciences; version 16) and Microsoft Excel 2007. In the statistical analysis, frequencies, percentage and average were determined.

## Results and discussion

A total of 317 patients were assessed in a 1 day free health camp organized by School of Health and Allied Sciences, Pokhara University, Nepal on May 18, 2013.

About 23 % of the patients were of age group 41–50 years (n = 69), followed by 16 % of the patients in the range of 31–40 years (n = 51). This showed that majority of patients were of working age (30–50 years). The most common problem was headache and fever and this symptomatic problem was followed by gastrointestinal disorders (Table 1). Studies conducted in Nepal have reported headache as one of the major physical complaints [5]. There was variation in the prevalence of symptoms, among different age groups, which might have been affected by the seasonal changes. Gastrointestinal problems were more prevalent in the age group 31–50 years which is similar to the findings of Sharma and co-workers [6]. In Nepal, 31–50 years is the active working age and the increased stress level at this age might have contributed to gastrointestinal problems [7]. Also, food and life style related factors might have led to this result which is supported by the fact that gastrointestinal disorder, is caused mainly by improper diet and habits, stress, spicy irritant food, oily foods, etc. [8]. Due to the lack of diagnostic facilities in the camp setting, diseases requiring diagnostic techniques were included less in number (e.g. cardiovascular disease). Some dental problems were also recorded, on the basis of symptoms.

**Table 1:** Age group of the patients versus disease

Age (years)	Symptomatic disorder	Respiratory disorder	Gastrointestinal disorder	Cardiovascular disorder	Inflammatory disorder	Ear problems	Gynaecological disorder	Skin disorder	Blood disorder	Urinary disorder	Dental problems	Total
≤10	6	8	8	0	0	0	0	5	0	0	1	28
11–20	11	3	17	0	0	2	1	6	3	0	0	43
21–30	11	6	14	0	2	1	3	2	0	1	0	40
31–40	19	4	18	0	0	0	7	2	0	1	0	51
41–50	25	5	25	0	7	0	5	1	0	1	0	69
51–60	19	2	5	2	3	0	1	1	0	1	0	34
61–70	12	0	8	0	2	1	0	1	0	0	0	24
>70	15	4	6	0	1	0	1	1	0	0	0	28
Total	118	32	101	2	15	4	18	19	3	4	1	317

Symptomatic disorder indicates headache and fever

**Fig. 1:**
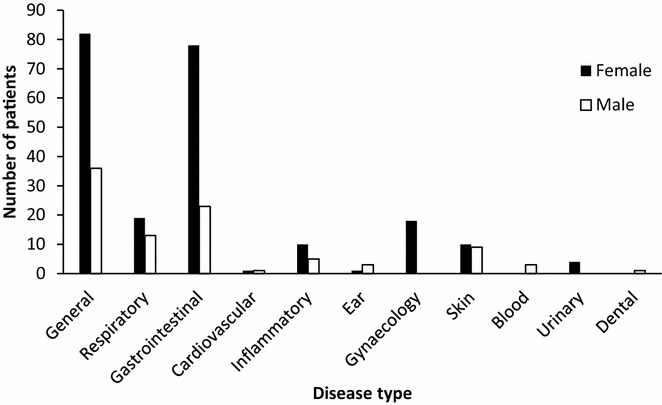
Gender-wise distribution of disease

Comparatively, higher number of women attended the health camp (Fig. 1). This might be due to the fact that most of the females are involved in house-hold activities in context of Nepal and can access to such health camps. On the other hand, male members visit city areas for various types of official works where they get easy access to health facilities. In Nepal, male literacy rate is 75.1 % and the female literacy rate is 57.4 % which might have also contributed to the result [7]. Additionally, it was observed that females attended health camps actively as compared to males. In comparison to other disease within each gender, gastrointestinal disorders were the most common diseases and prevalence of skin disorders was similar in both the genders (Fig. 1).

According to the 2011 census of Nepal, Chettris (16.6 %) and Brahmins (12.2 %) constitute higher percentage of the population [7]. Supporting this evidence our study showed that higher percentage of patients in the health camp were Brahmins (n = 144) and Chettris (n = 81). In these two major ethnic groups, gastrointestinal problems were the most common [6]. In contrast, the other ethnic groups were most commonly suffering from symptoms like headache and fever (Table 2).

**Table 2:** Ethnicity-wise distribution of disease

Disease	Brahmins	Chettri	Newar	Gurung	Magar	Dalit	Total
Symptomatic disorder	52	22	6	9	8	21	118
Respiratory disorder	11	10	2	2	1	6	32
Gastrointestinal disorder	55	30	1	3	3	9	101
Cardiovascular disorder	1	1	0	0	0	0	2
Inflammatory disorder	5	6	1	1	0	2	15
Ear problems	3	1	0	0	0	0	4
Gynaecological disorder	8	4	1	1	1	3	18
Skin problems	8	3	0	3	0	5	19
Blood disorder	0	1	1	0	0	1	3
Urinary disorder	0	3	0	0	0	1	4
Dental problems	1	0	0	0	0	0	1
Total	144	81	12	19	13	48	317

A total of 510 medications were prescribed. Among them, NSAIDs (29.41 %) were most commonly prescribed medication which is followed by antipeptic ulcer drugs (19.41 %) and vitamins and supplements (15.88 %) (Table 3). NSAIDs are the most commonly used medication in the World [9]. A combination of nimesulide and paracetamol was found to be the most preferred drugs by physician for fever. Patients who were prescribed with NSAIDs also received drugs to prevent gastritis [10]. Vitamins and supplements were also found to be widely prescribed medication. This might be because malnutrition in children and women is a major public health problem in most of the developing countries [11].

**Table 3:** Therapeutic category of drugs prescribed for different diseases

Disease	NSAIDs	Antibiotics	Antipeptic ulcer	Antiallergic and anticold	Antihypertensive	Antihelmintic	Vitamins and supplements	Antispasmodics	Antifungals	Bronchodilators	Others
Symptomatic disorder	89	4	13	3	0	4	29	0	0	0	0
Respiratory disorder	10	18	2	19	0	5	7	0	1	4	1
Gastrointestinal disorder	29	16	75	2	0	15	19	7	3	0	2
Cardiovascular disorder	1	0	0	0	2	0	0	0	0	0	0
Inflammatory disorder	15	1	6	0	0	0	3	0	0	0	0
Ear problems	0	4	0	2	0	1	2	0	0	0	0
Gynaecological disorder	2	16	1	0	0	0	12	0	8	0	2
Skin disorder	2	4	1	6	0	3	4	0	10	1	5
Blood disorder	1	0	0	0	0	3	3	0	0	0	0
Urinary disorder	1	4	1	0	0	1	2	1	1	0	0
Dental problems	0	1	0	0	0	0	0	0	0	0	0
Total	150	68	99	32	2	32	81	8	23	5	10

Supplements: calcium, iron, enzymes; others: antiseptic, oral rehydrating solution, contraceptives pills

The average number of drugs per prescription was 1.6 (Table 4), which was in accordance with the WHO standard [12]. In a similar study performed at one of the hospitals of Nepal, it was found that the average number of drugs per prescription was 2.5 [13]. This showed that the prescribing pattern differ from hospital to health camp. The involvement of pharmacists might have led to such consequence. The percentage of drugs prescribed by generic names was 96.8 % (Table 4), which is also close to the WHO standard (100 %) [12]. Although brand prescribing is a common practice in hospital settings of Nepal; in this study, doctors were found to prescribe by generic names. Also, the percentage of prescribed antibiotics was 21.4 % (Table 4), which falls within the WHO standard (20.0–26.8 %) [12]. This finding suggested that antibiotics were prescribed rationally. All the drugs prescribed were from the Essential Drug List (EDL) of Nepal (Table 4).

**Table 4:** Pattern of WHO prescribing indicators observed in the study

Prescribing indicators assessed	Total drugs/encounters	Average/percent	Standard derived or ideal
Average number of drugs per encountered	510	1.6	1.6–1.8
Percentage of encountered with antibiotic	68	21.4 %	20.0–26.8 %
Percentage of drugs prescribed by generic	297	96.8 %	100 %
Percentage of drugs from essential drug list	317	100 %	100 %

## Conclusion

Majority of the patients participated in the health camp were of working age group and female participants dominated the male participants in number. Among the studied symptoms and diseases, headache and fever were found to be the most prevalent. NSAIDs were the most commonly prescribed medications. In this study, the drug prescribing pattern complies with WHO recommended prescribing indicators. Since, health camp has a direct impact on disease prevention and treatment, similar studies related to the prescribing pattern are warranted in the future as well in order to assess the success of the health camp.
